# Challenges in Crohn’s Disease Management after Gastrointestinal Cancer Diagnosis

**DOI:** 10.3390/cancers13030574

**Published:** 2021-02-02

**Authors:** Claudio Fiorillo, Carlo Alberto Schena, Giuseppe Quero, Vito Laterza, Daniela Pugliese, Giuseppe Privitera, Fausto Rosa, Tommaso Schepis, Lisa Salvatore, Brunella Di Stefano, Luigi Larosa, Laura Maria Minordi, Luigi Natale, Giampaolo Tortora, Alessandro Armuzzi, Sergio Alfieri

**Affiliations:** 1 Digestive Surgery Unit, Fondazione Policlinico Universitario Agostino Gemelli IRCCS, 00168 Rome, Italy; carloalbertoschena@gmail.com (C.A.S.); giuseppe.quero@policlinicogemelli.it (G.Q.); vitolaterza.md@gmail.com (V.L.); fausto.rosa@policlinicogemelli.it (F.R.); sergio.alfieri@policlinicogemelli.it (S.A.); 2 Università Cattolica del Sacro Cuore of Rome, 00168 Rome, Italy; lisa.salvatore@policlinicogemelli.it (L.S.); giampaolo.tortora@policlinicogemelli.it (G.T.); 3 CEMAD-IBD Unit, Fondazione Policlinico Universitario Agostino Gemelli IRCCS, 00168 Rome, Italy; daniela.pugliese@policlinicogemelli.it (D.P.); gpp.privitera@icloud.com (G.P.); tommaso.schepis@gmail.com (T.S.); alessandro.armuzzi@policlinicogemelli.it (A.A.); 4 Unit of Medical Oncology, Comprehensive Cancer Center, Fondazione Policlinico Universitario Agostino Gemelli IRCCS, 00168 Rome, Italy; brunelladistefano89@gmail.com; 5 Department of Diagnostic Imaging, Oncological Radiotherapy, and Hematology–Diagnostic Imaging Area, Fondazione Policlinico Universitario Agostino Gemelli IRCCS, 00168 Roma, Italy; luigi.larosa@policlinicogemelli.it (L.L.); lauramaria.minordi@policlinicogemelli.it (L.M.M.); luigi.natale@policlinicogemelli.it (L.N.)

**Keywords:** Crohn’s disease, colorectal cancer, small bowel cancer, anal cancer, multidisciplinary approach

## Abstract

**Simple Summary:**

Crohn’s disease (CD) is a chronic inflammatory bowel disease affecting both young and elderly patients, involving the entire gastrointestinal tract from the mouth to anus. The chronic transmural inflammation can lead to several complications, among which gastrointestinal cancers represent one of the most life-threatening, with a higher risk of onset as compared to the general population. Moreover, diagnostic and therapeutic strategies in this subset of patients still represent a significant challenge for physicians. Thus, the aim of this review is to provide a comprehensive overview of the current evidence for an adequate diagnostic pathway and medical and surgical management of CD patients after gastrointestinal cancer onset.

**Abstract:**

Crohn’s disease (CD) is a chronic inflammatory bowel disease with a progressive course, potentially affecting the entire gastrointestinal tract from mouth to anus. Several studies have shown an increased risk of both intestinal and extra-intestinal cancer in patients with CD, due to long-standing transmural inflammation and damage accumulation. The similarity of symptoms among CD, its related complications and the de novo onset of gastrointestinal cancer raises difficulties in the differential diagnosis. In addition, once a cancer diagnosis in CD patients is made, selecting the appropriate treatment can be particularly challenging. Indeed, both surgical and oncological treatments are not always the same as that of the general population, due to the inflammatory context of the gastrointestinal tract and the potential exacerbation of gastrointestinal symptoms of patients with CD; moreover, the overlap of the neoplastic disease could lead to adjustments in the pharmacological treatment of the underlying CD, especially with regard to immunosuppressive drugs. For these reasons, a case-by-case analysis in a multidisciplinary approach is often appropriate for the best diagnostic and therapeutic evaluation of patients with CD after gastrointestinal cancer onset.

## 1. Introduction

Crohn’s Disease (CD) is a chronic, relapsing–remitting disease, which can affect the entire gastrointestinal tract with transmural inflammation [1,2,3]. It is characterized by a progressive course with damage accumulation and onset of complications [4,5,6]. CD patients have an increased risk of both intestinal and extra-intestinal cancers compared to the general population [7,8,9] and chronic inflammation has been identified as the main risk factor for cancerization [10,11,12]. 

In a prospective case–control study, Biancone et al. documented a general cancer incidence of 4.5 per 1000 CD patients over a period of two years [13]. A Danish historical cohort study, including about 50,000 Inflammatory Bowel Disease (IBD) patients over a period of more than 30 years, revealed a higher incidence of malignancies in patients affected by CD, with a Standardized Incidence Ratio (SIR) of 1.3 (95% CI 1.2–1.4), compared to the general population; notably, in the same study, CD patients also appeared to have an increased risk of cancer compared to ulcerative colitis (UC) patients [14]. 

With regard to gastrointestinal neoplasms, the most frequent IBD-related neoplasia, here including both patients with UC and CD with colonic involvement, is colorectal cancer (CRC) [11,15]. Besides CRC, recent evidence suggested that CD is associated with further gastrointestinal malignancies, in accordance with disease location and complications: small bowel cancer for ileal involvement, and anal and perianal cancer (typical of patients with chronically active perianal disease) [7]. 

Gastrointestinal cancers are currently one of the main causes of disease-related death in CD patients [16]. In the Florence IBD study, 920 IBD patients were prospectively followed-up over a period of more than 30 years: in this cohort, CD patients were reported to have an increased overall mortality (Standardized Mortality Ratio (SMR) of 1.79, 95% CI 1.39–2.27) and an increased risk of dying from cancer (SMR of 1.85, 95% CI 1.22–2.69) [17] compared to the general population.

CD treatment is based on the use of immunosuppressors, which do not seem to increase the risk of developing cancer, beside a slightly increased risk of lymphoproliferative and cutaneous malignancies associated with thiopurines and tumor necrosis factor (TNF) antagonists [18]. However, immunosuppressive therapies are not recommended in patients with current cancer, and a 2- or 5-year disease-free interval is usually required before they can be safely started after a diagnosis of cancer [9]. This makes CD management particularly challenging in patients who receive a cancer diagnosis. Furthermore, it has been documented that some oncologic therapies (such as hormonal therapy and check-point inhibition) can exacerbate gastrointestinal symptoms [19]. 

Accordingly, the multidisciplinary approach, requiring the conjunct efforts of gastroenterologists, surgeons, radiologists and oncologists, is crucial for CD patients who receive a diagnosis of gastrointestinal cancer. Thus, the aim of this review is to summarize the current knowledge on CD-related gastrointestinal malignancies, including the point of view of all specialists who concur to the management of the patient on three main key areas:

- Diagnosis of gastrointestinal cancers in CD patients;
- Medical and surgical treatment of gastrointestinal cancers in CD patients;
- Treatment of CD after the diagnosis of cancer.

## 2. Epidemiology and Pathogenesis of CD-Related Gastrointestinal Cancers

### 2.1. Colorectal Cancer

#### 2.1.1. Epidemiology

The connection between IBD and CRC was identified in the first decades of the last century, after the report by Crohn and Rosenberg [20]. The modern management of CD, based on strict endoscopic surveillance, a patient-tailored medical treatment and a more aggressive surgical approach, has progressively brought to a slight decrease in CRC onset in CD patients [21,22]. For instance, Canavan et al. and Von Roon et al. reported a pooled SIR of 2.5 (95% CI 1.3–4.7) and 2.4 (95% CI 1.56–4.36), respectively [23,24], while a lower value was shown by Jess et al. and Lutgens et al., with an SIR of 1.9 (95% CI 1.4–2.5) and 1.7 (95% CI 1.01–2.5), respectively [25,26]. 

In comparison to the general population, CD-related CRC usually appears at an earlier age (40–50 years vs. 60 years) [27], with a higher incidence in case of CD onset before 15 years of age [14]. It generally presents as a mucinous or signet-ring histotype with a higher histological grade and it is frequently associated to synchronous or metachronous lesions [28].

Although CD patients are evidently more prone to develop CRC as compared to the general population, a recent meta-analysis [29] and a case-control study [30] showed similar overall survivals when CD-related and sporadic CRCs were compared. More specifically, Olén et al. reported a mortality of 0.47 and 0.31 per 1000 person-years for CD-related and sporadic CRC, respectively, during their study period of almost 50 years [31]. However, the sub-analysis of patients suitable for current surveillance programs revealed an increased mortality rate in case of CD onset before 40 years of age, colonic involvement and coexisting primary sclerosing cholangitis (PSC) [31].

#### 2.1.2. Pathogenesis and Risk Factors

Several studies showed different etiopathological mechanisms for sporadic and IBD-related colonic neoplasms. Sporadic CRC arises from premalignant adenomatous polyps as a result of the well-known adenoma–carcinoma sequence with a well-established pattern of genetic mutations. Conversely, in IBD-related CRCs, a key role is played by the chronic inflammatory damage associated to an altered microbiota [8,10,32]. In genetically susceptible patients [33], these factors may lead to the inflammation–dysplasia–carcinoma sequence instead of the classic adenoma–carcinoma sequence [34,35,36]. 

With regard to the risk factors for CRC or dysplasia onset in patients affected by CD, the duration, severity and extent of the colonic inflammation have been recognized as the main predisposing features [5,21,37]. Indeed, patients with eight or more years of CD-related colitis are more prone to CRC development, highlighting the need for an ad hoc surveillance program, especially after this time elapse [31,36,38]. In addition, the early onset of CD, in particular before 15 years of age, and the detection of new colonic strictures in longstanding colitis have been recognized as further risk factors [14,39]. Although the exact connection is still not completely clear, concomitant diagnosis of PSC, a family history of CRC and the penetrating pattern of CD have been identified as playing an additional key role in the onset of CD-related CRCs [13,40]. 

### 2.2. Small Bowel Cancer

#### 2.2.1. Epidemiology

Small bowel carcinoma (SBC) can be considered a rare complication of CD, with an estimated cumulative risk of 2.2% after a 25-year clinical history of regional ileitis [41]. As compared to the general population, CD is associated with nearly a 10-fold increased risk of SBC, with a similar incidence between males and females, but a significantly higher rate in patients aged 60 years old or over [42]. In terms of the time elapse of the major incidence, the first year after CD diagnosis relates to a 100-fold increased probability of SBC development. Conversely, a significant drop to a five-fold higher risk is reported from the second year after diagnosis onwards [42]. In terms of tumor location, CD-related SBC more frequently arises in the ileum, as compared to sporadic SBC that is more evenly distributed between the jejunum and ileum [43]. When adequately and promptly diagnosed, CD-related SBC has shown a 5-year survival rate between 35% and 43% [43,44,45]. This data did not significantly differ from the sporadic manifestation of SBC [44,45]. Moreover, a survival analysis stratified by stage showed similar rates for stages I–IV of CD-SBC as compared to the sporadic SBC [43,46]. 

#### 2.2.2. Pathogenesis and Risk Factors

Few retrospectives series specifically analyzed the correlation between CD and SBC. A chronic penetrating disease, immunomodulators and steroids exposure, stricturoplasties and small bowel by-pass loops are currently recognized as risk factors [47]. Moreover, Lashner et al., in a case–control study, found 6-mercaptopurine use and proximal small bowel disease as significantly associated to SBC in patients with CD [48]. Conversely, the results published in 2004 by Solem et al. did not recognize any specific risk factor, although the use of salicylates was identified as protective against SBC onset [49]. In a larger cohort multicenter study, the surgical resection of the tract of ileum affected by CD as well as the prescription of salicylates for more than two years resulted in markedly reducing the risk of SBC development [50]. 

Although no studies specifically analyzed the physio pathology of the correlation between CD-induced inflammation and higher risk of SBC onset, the current hypothesis is based on the potential evolution from inflammation to dysplasia. Indeed, epithelial dysplasia was found in the proximity of SBC in 7 out of 8 patients in a pathological study presented by Sigel et al. [51]. A further confirmation of this finding was also given by Svrcek et al., who demonstrated a dysplastic lesion adjacent to CD-associated SBC, with several molecular similarities to colitis-associated colorectal cancer [52]. Based on these data, although no effective screening methods for SBC are currently present, the findings of preneoplastic changes could hypothetically guide the decision-making in CD patients. 

Similarly, the protective effect of salicylates is not yet fully understood. Indeed, salicylates prescription could reflect a milder disease activity and, thus, would not have a preventive role per se. On the other hand, salicylates (especially for formulation with release in the small bowel) may reduce the local inflammatory response, leading to a decreased risk of SBC [50]. Conversely, the association between immunosuppressive drugs and increased risk of SBS might be attributable to the higher inflammatory burden that characterize CD patients who require advanced therapies. 

### 2.3. Anal Cancer

#### 2.3.1. Epidemiology

The risk of anal cancer is increased in patients with CD, mainly in the form of fistula-associated anal carcinoma (carcinoma arising from perianal fistula) [23,25]. Once thought to be rare, anal carcinoma in CD is gaining a growing attention due to increasing reports in the literature [53,54,55,56,57,58,59,60,61]. On the other hand, it is difficult to define its exact incidence because of the lack of population-based studies. As a consequence, there is no unanimous consensus on its diagnosis, surveillance and overall management.

A large case–control study by Beaugerie et al. [32] reported an incidence of 0.38 and 0.26 per 1000 patients/year for adenocarcinoma (ADC) and squamous cell carcinoma (SCC), respectively, after prospectively monitoring more than 2900 patients with a history of active or previous anal/perianal CD. Moreover, a long-standing perianal fistulas history (>10 years) seems the main risk factor for the onset of anal malignancies in CD, as confirmed by other studies [62,63,64]. 

Similar results were shown by a systematic review, reporting ADC as the most frequently reported histotype among patients with anal fistulizing CD, followed by SCC [65]. 

Patients with fistula-associated carcinoma in CD have a poor prognosis, with a 5-year survival rate of 37% compared with 60% in the general population [66]. 

In a systematic review analyzing 65 ADC cases, 30 patients died after a median follow up of 20.5 months, with an overall survival rate at 1, 3 and 5 years of 88%, 54% and 26%, respectively [57]. Prognosis for SCC is dismal as well. A literature review [59] analyzed 17 patients with a 3.5-year median follow up: 8 patients died of primary cancer (47%), 7 of which in the first year, while the median survival among the survivor was 5 years (0.25–25 years range). 

The reason behind this poor prognosis seems to be the delay of diagnosis due to the similarity of the symptoms with the ones of severe perianal disease, such as pain, recurrent abscesses and intractable fistulas. Accordingly, any change in the severity of symptoms, such as a new onset of incontinence, new or different pain, bloody mucus discharge and obstruction, should be considered as a warning sign for the possible onset of cancer. 

#### 2.3.2. Pathogenesis and Risk Factors

The pathogenesis of fistula-associated anal carcinoma in CD likely follows the same paradigm of longstanding colitis, triggered by chronic inflammation and mucosal hyperplasia followed by dysplasia and carcinoma [58]. It is not clear if ADC arises from the epithelial lining of the fistula or from rectal-type mucosa cells migrating to the perianal fistula [64]. Regarding SCC, the same authors reported the presence of high-risk human papilloma virus (HPV) in all the patients with SCC and IBD, confirming the link between HPV infection history and perianal fistula-associated SCC also in IBD patients [64]. 

The impact of immunosuppressant and biologic drugs on anal cancer onset in CD still remains unclear: on one hand, the reduction of inflammatory activity could lower the cancer incidence; on the other hand, a suppressed immune system could also lead to increasing the risk of carcinoma [64,65,67,68], but these results were not supported by subsequent studies [63,69,70,71]. Further studies are needed to clarify these aspects. 

## 3. Diagnosis and Imaging

Endoscopy is still today the cornerstone of the diagnosis and surveillance of gastro-intestinal malignancies. However, radiological imaging is progressively gaining importance for an appropriate diagnosis and adequate tumor staging. 

Computed tomography (CT) and magnetic resonance (MR) may contribute to the detection of intestinal cancers in patients with IBD. These techniques provide panoramic and multiplanar images, and allow evaluation of mural involvement, mesenteric extension, presence of pathological lymph nodes, metastases and complications such as perforation, bleeding and occlusion. 

The preference of MR over CT is based on available resources and an absence of radiation exposure. This is especially important in radiosensitive patients with IBD, often developing symptoms in adolescence and in young adulthood, who undergo multiple studies over a lifetime. Moreover, the choice of the best technique to use depends on which intestinal tract is affected: small bowel, colon or rectum and anus.

### 3.1. Colorectal Cancer

The endoscopic surveillance for CRC is a well-established procedure that allows the detection of malignant and pre-malignant lesions (e.g., dysplasia), guaranteeing a potential reduction in CRC-related mortality [72]. Indeed, a large retrospective study on 6823 IBD patients reported a significantly lower incidence of CRC in the subgroup of patients undergoing surveillance colonoscopy [73]. According to the National Institute for Health and Care Excellence (NICE) and the European Crohn’s and Colitis Organization (ECCO), the first screening colonoscopy should be performed 8 years after IBD symptoms onset [38,74]. 

The timing of the surveillance should be based on a risk stratification [75]. Patients with even just one of these following characteristics, including colonic strictures, personal history of dysplasia within the previous 5 years, PSC, extensive colitis with severe inflammation and a family history of CRC diagnosed before the age of 50, are considered at high risk and should undergo an annual colonoscopy. Extensive colitis with mild or moderate inflammation, pseudopolyps and family history of CRC diagnosed after the age of 50 are considered intermediate risk factors, requiring surveillance every 2–3 years. Patients who do not fulfill high and intermediate risk factors can have a screening colonoscopy every 5 years. 

An extremely meticulous bowel preparation is crucial to allow the endoscopic detection of nonpolypoid mucosal lesions [76]. In addition, an accurate colonoscope withdrawal is crucial and a time longer than 6 minutes has been demonstrated to increase the probability of mucosal malignancy and pre-malignancy detection [77]. Moreover, a high-definition endoscopy with chromoendoscopy with targeted biopsies of any visible lesions should be performed during surveillance [78]. A metanalysis by Feuerstein et al. reported the superiority of chromoendoscopy over standard white-light endoscopy in dysplasia detection [79]. If chromoendoscopy is not available, random biopsies should be performed obtaining 4 samples every 10 cm [75]. 

Nowadays, new endoscopic tools are available for the detection of colonic pre-malignancies, such as virtual chromoendoscopy, autofluorescence imaging, confocal laser endomicroscopy and endocytoscopy [80]. However, their role in daily clinical practice is not yet established. 

Although the diagnosis of CRC is usually made with colonoscopy, radiological imaging could first suggest the diagnosis on the basis of CT findings.

A preoperative CT is useful for planning surgery or radiation therapy, particularly when involvement of adjacent organs and/or metastases are identified. In addition, preoperative CT provides a baseline exam for comparison during the postoperative period and it is the best modality for detection of local recurrence after surgery.

Some authors have proposed the distension of the colon via a rectal tube in patients with CD, in order to better evaluate the colonic involvement [81,82]. Neutral agents (water) or negative agents (air) can also be easily administered via the rectal tube and provide excellent contrast for colonic imaging [83,84,85]. Gazelle et al. performed CT after administration of a water enema, demonstrating effectiveness in staging colorectal cancers; this technique may improve the ability of CT to demonstrate the degree of tumor invasion of the bowel wall and the extension into the extra-visceral space [86]. Administration of intravenous contrast medium is mandatory for complete staging. 

**Figure 1:**
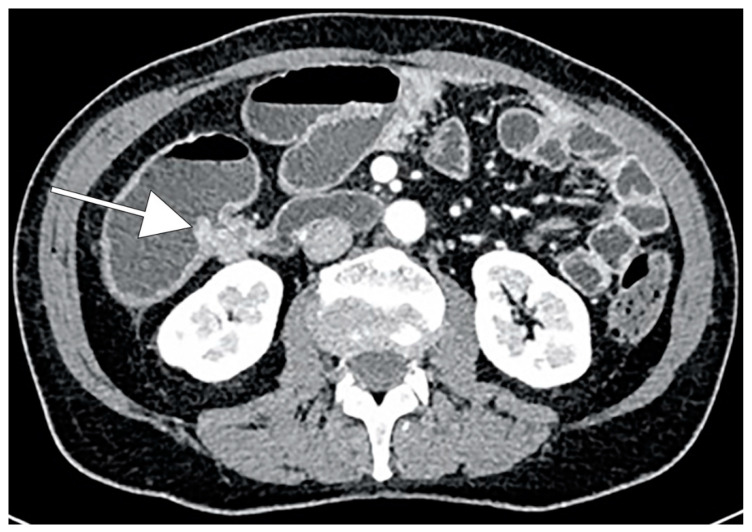
CT scan demonstrating wall thickening colorectal cancer (CRC) of the right colon (white arrow).

CRC typically appears as a discrete soft-tissue mass that narrows the colonic lumen. Large masses may have central necrosis, evidenced by CT as a soft-tissue mass with central low attenuation. When the central area shows air attenuation, this appearance may look like an abscess. In addition, a significant percentage of CRCs appear as focal wall thickening without luminal narrowing (Figure 1). CT can easily identify complications of primary colonic tumors, such as obstruction, perforation and fistulas. The sensitivity of CT in detection of bowel obstruction (small bowel and colon) and perforation is high, ranging between 90% and 94% [87].

### 3.2. Small Bowel Cancer

The current diagnostic techniques for CD-related SBC present several limitations. Indeed, diagnosis is generally made during laparotomy or during the pathological examination of the specimen [44]; this may justify the high rate of metastatic disease at the time of diagnosis (30% to 35%) with a node-positive disease encountered in 55% of patients who undergo surgery [88]. Although an endoscopic screening was proposed, the current techniques demonstrated poor results due to frequent impassable strictures and incomplete visualization of the small bowel [46,89]. 

The endoscopic techniques available for small bowel cancer diagnosis are upper endoscopy, enteroscopy and wireless video capsule endoscopy (VCE) [90]. The upper endoscopy allows the exploration of the first tract of the small bowel and may be useful in detecting duodenal or proximal jejunal tumors. Enteroscopy allows the visualization of the all-small bowel surface with a push or double-balloon technique, giving also the opportunity of sample collection and therapeutic procedures [91]. This technique could be useful in patients with suspicious small bowel stricture in order to perform a differential diagnosis between inflammatory stricture and small bowel cancer. Conversely, VCE is a less invasive procedure requiring the ingestion of a video capsule that records during the passage through the gastrointestinal tract, but it does not allow to perform biopsies and should be avoided in patients with known strictures [92]. 

**Figure 2:**
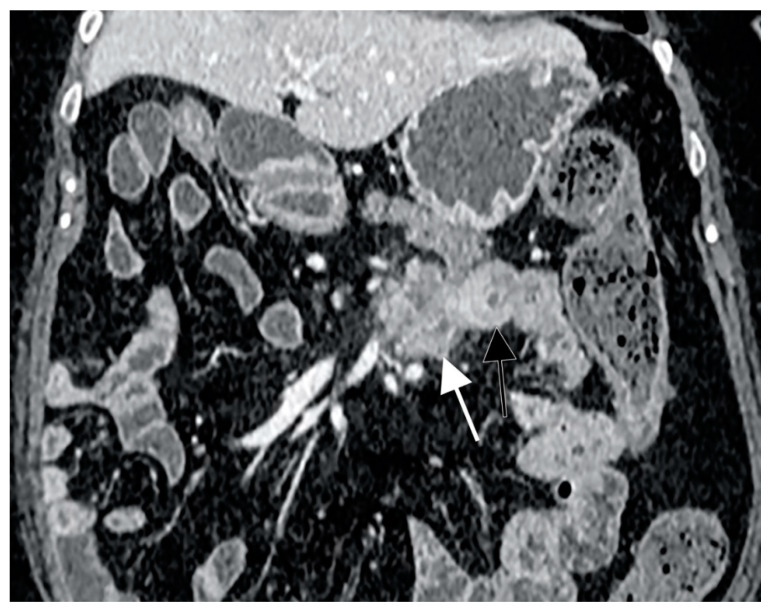
CT scan representation of small bowel carcinoma (SBC). White and black arrows show heterogeneous attenuation and moderate enhancement after contrast medium injection.

With regard to radiological exams, the primary requirements of small bowel imaging are the visualization of the whole intestinal tract and an adequate bowel distension. In order to achieve them, CT enterography (CTE) and MR enterography (MRE) are performed using large volumes of oral contrast. In 2013, Soyer et al. performed a meta-analysis showing a sensitivity and specificity of CT-enteroclysis in the detection of small-bowel cancers of 92.8% and 99.2% (95% CI 94.2–99.9%), respectively [93]. Subgroup analysis revealed that for small-bowel preparation, more than one imaging pass and larger volumes (≥2 L) of oral contrast agent did not improve the cancer detection rate. Incomplete distension, functional invaginations or intestinal spasm were shown to give false-positive interpretations [94]. SBC appears as a solitary soft-tissue lesion or ulcerated mass with circumferential or eccentric luminal narrowing, usually involving a short segment and causing partial or complete bowel obstruction [95]. CT typically shows heterogeneous attenuation and moderate enhancement after contrast medium injection (Figure 2), and it is able to detect metastases that can occur in local lymph nodes, liver, peritoneal surfaces and ovaries. However, lymph node enlargement is not as marked as in lymphomatous disease [96]. 

### 3.3. Anal Cancer

A prompt examination under anesthesia with biopsy remains the most reliable diagnostic step and it is warranted in any case of clinical suspicion [54,55,56,57,97]. However, imaging techniques also have shown convincing results in the assessment of the disease. MR imaging permits an excellent evaluation of the anal canal, sphincter complex and local lymph nodes, due to its high contrast resolution. The reported sensitivity of MR for anal cancer diagnosis is 90–93%; its limit may be the visualization of small superficial lesions, which can be identified by endoanal ultrasound with 100% sensitivity [98,99,100,101].

**Figure 3:**
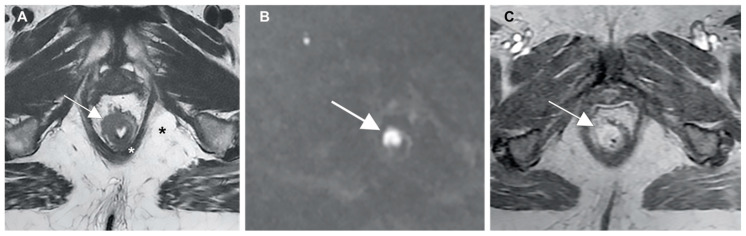
(**A**) Anal cancer captured using MR imaging, showing (white arrows) a higher signal intensity than the mucosal and submucosal layers; (**B**) restriction of proton diffusion at the DWI sequence; and (**C**) post-contrast enhancement after gadolinium injection.

Preoperative staging techniques for anal cancer allows detection of sphincter involvement, extra-parietal spread, mesorectal fascia infiltration and lymph node enlargement. In locally advanced cancers, MR imaging can establish the relationship between the tumor and the adjacent pelvic structures. The tumor has an intermediate signal intensity between the fat tissue (high signal intensity) and muscular layer (low signal intensity). Moreover, its signal intensity is higher than that of the mucosal and submucosal layers (Figure 3A), and the DWI sequence shows restriction of proton diffusion (Figure 3B). It shows avid post-contrast enhancement after gadolinium injection (Figure 3C) and usually has an infiltrative or lobulated intraluminal growth pattern [102,103,104,105]. 

## 4. Medical Management

The diagnosis of cancer in patients with IBD offers numerous challenges to physicians. Each case requires a comprehensive assessment by a multidisciplinary team, involving oncologists, radiotherapists and any other related healthcare advisor [106]. Offering the appropriate treatment for concomitant cancer has to be the priority for increasing the survival chances. However, previous studies have shown that the comorbid IBD can influence the choice and/or the timing of oncological treatments and accordingly the percentages of success [107,108,109]. Moreover, with regard to CRC, a diagnosis in patients on long-term immunosuppressive therapy seems to be associated with similar tolerance to surgery, but also with a worse long-term outcome in terms of disease-free survival and overall survival, as compared to not-immunosuppressed ones [110].

Several treatment options for cancer, including cytotoxic/hormone drugs and radiation treatments, can also induce IBD flares, causing further delay or suboptimal cancer treatment [19,111,112]. 

On the other hand, after recovering from cancer, patients could require advanced immunosuppressive therapies for uncontrolled IBD, being potentially exposed to an increased risk of cancer recurrence. The length of cancer-free survival before immunosuppressive agent introduction and the choice among different molecules should be tailored to the individual patient and shared in a multidisciplinary setting [106].

### 4.1. Management of IBD during Cancer Treatment

As previously stated, according to the type and stage of cancer, some neoplasms may require a single treatment protocol and others, instead, a multimodal approach (e.g., a combination of surgery and chemotherapy). Accordingly, physicians should enhance IBD control in order to favor the completion of selected cancer treatments. The use of immunosuppressive therapies with a potential carcinogenic effect, including thiopurines, calcineurin inhibitors and biological drugs, is not recommended, unlike mesalamine compounds and steroids, which are widely considered safe and preferred for their effects on cancer-related symptoms [18,113]. However, for selected patients with advanced cancer and severe IBD relapse, the use of anti TNF-alpha drugs could be acceptable in order to achieve a better control of symptoms and an improvement of quality of life [114,115]. To date, there are no studies exploring the safety and effectiveness of non-anti TNF-alpha biological drugs for patients with current cancer. Conversely, vedolizumab, like infliximab, represents the second-line therapy after steroids failure, for the treatment of immune checkpoint-inhibitor enterocolitis [116].

The impact of oncological treatments on IBD has been poorly investigated [19,112,117]. The largest cohort comes from Axelrad et al., who reported data of 84 patients with IBD treated with chemotherapy and/or hormonal therapy for solid malignant extra-intestinal neoplasm. Of note, a small number of them (12 on thiopurines and 3 on anti TNF-alpha drugs) maintained their immunosuppressive therapy through their cancer treatment. Among the 69 patients who started cancer therapy while IBD in remission, 17.4% experienced a relapse. Hormonal therapy alone (HR: 11.04; 95% CI 1.22–99.85) or in combination with cytotoxic chemotherapy (HR: 9.71; 95% CI 1.16–81.08) increased the risk of IBD reactivation. Conversely, cytotoxic chemotherapy induced IBD clinical remission among 66.7% of patients who started cancer therapies with active IBD [19].

Abdominal or pelvic radiation treatment has always raised several concerns among oncologists due to the potential risk of complications, including IBD flare and radiation-related gastrointestinal injury, even though based on old and sometimes conflicting data [118,119,120]. However, more recent studies, reporting data on modern techniques, such as intensity-modulated radiation therapy (IMRT), seem to minimize the absolute risk of complications and break down the axiom that IBD is an absolute contraindication for radiation treatment [121].

### 4.2. Active IBD in Patients with Previous History of Cancer

Mesalamine compounds and steroids are recommended as first-line medical therapies to manage IBD flares in the first five cancer-free years. However, in case of patients with severe IBD activity, unresponsive to those drugs and not suitable for surgery, the use of immunosuppressants could be considered case-by-case in a multidisciplinary setting [9]. There are two critical elements in this decision-making process: (1) estimating the risk of recurrence of each different neoplasia after immunosuppressive therapy; and (2) estimating the risk of promoting cancer recurrence of each specific IBD drug.

For the first point, the experience coming from transplant recipients, who receive life-long and multiple immunosuppressive therapies, have allowed to stratify neoplasia in low (<10%), intermediate (11–25%) and high risk (>25%) of recurrence after starting immunosuppression [122]. Furthermore, the length of cancer-free survival before immunosuppressive agent introduction in terms of years has been shown to be inversely related to the risk of recurrence [123]. Notably, several anti-rejection drugs, such as tacrolimus, mycophenolate mofetil or sirolimus, are not in use for treating CD.

According to this classification, CRC is considered at intermediate risk of recurrence, requiring at least 5 years of cancer-free survival before immunosuppressive agent introduction [9].

However, the comprehensive estimation of cancer recurrence from oncologists does not rely only on this mere classification, but takes into account the stage, grading and molecular features of each neoplasia and all available data on therapeutic effectiveness [18].

With regard to the risks related to IBD drugs, no relevant safety information can be retrieved from clinical trials, since a previous history of cancer, except for non-melanoma skin cancers, is a standard exclusion criterion for most of those with biologics.

A meta-analysis of 16 observational studies by Shelton et al., including 11,702 cases, explored the risk of cancer recurrence among patients affected by immune-mediated diseases (IBD, rheumatic disease and psoriasis) and treated with thiopurines, methotrexate, anti TNF-alpha or a combination of them (anti TNF-alpha plus immunomodulator). No therapy-related significant adjunctive risk of cancer recurrence emerged in both the overall cohort and in each subgroup stratified for type of immune-mediated disease. The median time from diagnosis of index cancer and the immunomodulators start date was 6 years, but no differences were found in terms of the pooled incidence of new or primary cancer when the therapy was started within or after this time point [124].

Recently, data on the safety of vedolizumab in patients with a history of previous cancer have been reported. In particular, 96 patients exposed to vedolizumab (median time from cancer of 3.9 years, range 0.1–43) were compared to 184 and 183 patients (median time from cancer of 1.3 years, range 0–38) exposed to anti TNF-alpha or to no immunosuppressive therapy, respectively. Notably, 41% of patients treated with anti TNF-alpha never stopped their biological therapy after the diagnosis of cancer. Gastrointestinal type accounted for 7% of the index cancers. Both treatments with vedolizumab (HR 1.38; 95% CI 0.38–1.36) and infliximab (HR 1.03; 95% CI 0.65–1.64) seem not to increase the risk of new or recurrent cancer, nor after adjusting for confounding factors, such as the risk of recurrence of each neoplasia according to the classification mentioned above [122] and the time to initiate biologic agents after cancer diagnosis [125].

## 5. Surgical Management

### 5.1. Colorectal Cancer

As for sporadic CRCs, colorectal resection with lymphadenectomy represents the gold standard for the treatment of resectable CD-related CRCs [126,127,128]. However, disease localization and cancer stage, patients’ characteristics, comorbidities, expected quality of life and CD natural history are key factors that should be taken into account for indication to surgery. Furthermore, the 80% lifetime risk of intestinal resection in CD patients might significantly increase the complexity of the surgical procedures and influence the surgical treatment choice [129,130]. This inevitably reflects in the need of a tailored surgical treatment for each patient, on the base of a multidisciplinary discussion between gastroenterologists, oncologists and colorectal surgeons.

Total proctocolectomy is the gold standard of treatment for CD-related CRC in patients defined fit for surgery, due to the high risk of synchronous dysplasia or cancer [126,127,128,131,132,133]. However, total colectomy or segmental colectomy are possible alternatives, especially in case of patients with multiple comorbidities [134,135,136]. In these cases, a strict postoperative surveillance should be mandatory.

#### 5.1.1. Total Proctocolectomy (TPC)

TPC should be considered the standard of care for fit patients with CD-related CRC or dysplasia, in particular in case of concomitant involvement of the rectum. The rationale of this radical approach is based on the assumption that the colorectal excision could prevent synchronous or metachronous disease [9,110,131,136]. According to Kiran et al., 14.3% of patients who underwent segmental colonic resection for CD-related CRC developed a metachronous cancer in the spare colon [110]. A study conducted at Mount Sinai Medical Center [131] revealed that 40% of patients who underwent a segmental colonic resection and 35% who had subtotal colectomy for CD-related CRC subsequently developed metachronous neoplasia after a mean period of 6.8 years. Moreover, the authors reported a similar incidence of metachronous disease comparing subtotal colectomy with segmental resection, highlighting the high risk of new disease, whether in the remaining colon or rectum. 

An end ileostomy is usually performed after TPC, especially for those patients with perianal CD involvement, while an ileal pouch anal anastomosis (IPAA) could be considered as an option only in a limited percentage of selected patients with normal sphincter function and without perianal or small bowel disease [137,138,139,140]. This choice is driven by the high risk of pouch failure, pelvic sepsis and CD onset on the IPAA, when performed [141,142,143,144,145,146].

#### 5.1.2. Total Abdominal Colectomy

Total abdominal colectomy with end ileostomy or with ileorectal anastomosis may be considered as a valid option in patients with no involvement of the rectum. The main benefit of this surgical procedure is the avoidance of sexual or urinary dysfunctions related to the pelvic dissection [147,148]. Specifically, total abdominal colectomy with ileorectal anastomosis may be a treatment option in case of adequate continence and sphincter function, while it should be avoided in case of high-dose steroids therapy for more than six weeks, due to the increased risk of anastomotic leakage or infectious complications [149,150].

However, the subsequent risk of rectal cancer should not be underestimated [69], thus implying the need for specific surveillance programs in this set of patients [151,152,153]. Derikx et al., in their systematic review and metanalysis, evidenced a pooled carcinoma prevalence of 2.1% in CD patients with rectal stump and permanent ileostomy, and of 0.7% in those with ileorectal anastomosis [154]. A recent retrospective cohort study of IBD patients highlighted an incidence rate of rectal stump cancer and high-grade dysplasia of 4.8 per 1000 patients-years of follow up [155].

#### 5.1.3. Segmental Colectomy (SC)

SC for CD-related CRC finds limited indications. It is generally reserved to well defined solitary lesions, affecting a limited segment of colon, stable over the years, and to those patients with multiple comorbidities, for whom a more aggressive surgical procedure could be detrimental. Inevitably, despite the lower risk of stoma formation, SC is linked to a higher incidence of metachronous lesions over the years, and to a higher risk of postoperative complications as compared to total abdominal colectomy [156].

### 5.2. Colorectal Dysplasia in Crohn’s Disease

A major dilemma is currently represented by the treatment of CRC premalignant lesions, namely low- to high-grade dysplasia. Given the paucity of specific data, the current recommendations are based on previous experiences on UC [36]. Early studies documented a cumulative prevalence of CD-related dysplasia from 2% to 5% [157,158], with an estimated 25-year risk up to 25% [159,160]. Interestingly, a histopathological review study of surgical specimens of Crohn’s colitis reported the presence of carcinoma contiguous to high-grade dysplasia in 56% to 100% of cases, while the incidence of dysplasia distant from cancer ranged between 38% and 41% [51,161,162]. 

Although the ECCO guidelines [126,163] and American Society of Colon and Rectal Surgeons (ASCRS) guidelines [127] define the detection of dysplasia as potential indications to surgery, multiple factors, especially the macroscopic and microscopic characteristics of the lesions, should be taken into consideration for the decision making. 

The microscopic classification of dysplasia was firstly reported in 1983 by Riddell et al., who recognized four main categories: no dysplasia, indefinite dysplasia and low-grade and high-grade dysplasia [164]. Macroscopically, lesions are currently categorized as invisible or visible. These latter ones have been further classified as polypoid (sessile or pedunculated) or nonpolypoid (depressed, lift or slightly elevated) lesions [165,166]. 

The detection of dysplasia during surveillance colonoscopy does not represent an indication to surgery per se. Indeed, for visible low- to high-grade dysplastic lesions, in the absence of any other lesion of the colon or in the nearby flat mucosa, the endoscopic resection should represent the treatment of choice [36,127,160]. Although little evidence is currently present in the literature on the effectiveness of the endoscopic submucosal dissection (ESD) in CD patients, this approach has demonstrated to provide a high rate of complete resection when performed by an expert endoscopist [167]. Technically, the en-bloc resection is preferred over the piecemeal excision in order to obtain a correct pathologic evaluation of the completeness of the resection. Moreover, a tattoo on the site of the endoscopic resection is highly recommended in order to simplify the endoscopic surveillance, and biopsies in the flat mucosa adjacent the site of resection should be acquired with the aim to analyze the nearby mucosa status [168]. 

On the counterpart, a more radical treatment is indicated for visible dysplastic lesions not endoscopically resectable, or in case of multifocal dysplasia or also in case of dysplasia in the nearby flat mucosa. In these cases, total colectomy with or without proctectomy is advised rather than segmental colectomy, due to the high risk of coexisting dysplasia in the remaining colon [127]. Indeed, Kiran et al. reported a 36% risk of high-grade dysplasia and a 45% risk of CRC on the surgical specimen in case of a preoperative diagnosis of low-grade or high-grade dysplasia, respectively [136].

**Figure 4:**
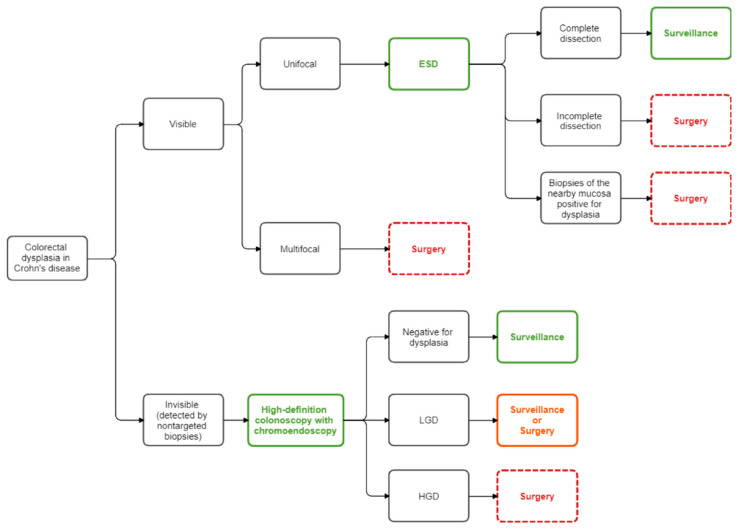
Flow diagram for the management of colorectal dysplasia in Crohn’s disease. ESD: endoscopic submucosal dissection; HGD: high-grade dysplasia; LGD: low-grade dysplasia.

As opposed to sporadic and family-hereditary forms, CRC in patients affected by IBD may also arise from invisible dysplasia related to a “field change effect” on the colonic mucosa [36,169]. In case of multifocal invisible dysplasia or invisible dysplasia in two consecutive high-definition colonoscopies with chromoendoscopy, independently of the grade of dysplasia detected (low or high-grade), total colectomy or total proctocolectomy is recommended [36,127,170]. However, controversy is still present in case of detection of unifocal invisible low-grade dysplasia. Indeed, in selected patients, a strict endoscopic surveillance may be appropriate as an alternative to resection [36,127,171,172]. Figure 4 summarizes the current management of colorectal dysplasia.

### 5.3. Small Bowel Cancer

Independently of the tumor location, surgery performed as a radical resection represents the mainstay of treatment. According to the third ECCO pathogenesis scientific workshop, surgical resection is highly encouraged when low- to high-grade dysplasia is detected at the endoscopic small bowel biopsy in the area of inflammation [47]. However, CD-related SBC is frequently diagnosed intraoperatively, and almost half of the cases are diagnosed only at the histological analysis of the surgical specimen [41,173,174,175]. Accordingly, preventive surgical resection has been advocated in order to remove the chronically inflamed segments, especially in those patients followed up for more than 10 years and in case of dysplasia on endoscopic biopsies [44,50]. Surgery for CD-related SBC is the same as for de novo small bowel tumors. More specifically, wide resection of the small bowel with the corresponding mesentery and lymph nodes is the treatment choice in case of jejunum/ileal location. The number of lymph nodes to be retrieved in order to define resection as oncologically radical is still matter of debate [176,177]. Although a threshold is not yet set, 10 nodes have been defined as the minimum number for an adequate resection. In case of SBC in the distal ileum, a right colectomy is indicated. Pancreaticoduodenectomy is, instead, indicated for lesions located in the second or third portion of the duodenum. Duodenal segmental resection was proposed as an alternative treatment to pancreaticoduodenectomy. Despite the advantages in terms of overall survival as compared to the Whipple procedure reported by Lowell et al. [178], these data were not confirmed by the more recent experience of the Cleveland Clinic [179].

### 5.4. Anal Cancer

Fistula-associated anal carcinoma in perianal CD often presents at a locally or systemically advanced stage due to delays in diagnosis. Since the low incidence of this disease and the consequent lack of conclusive evidence, no specific treatment guidelines are currently available. 

A systematic review of 23 studies including 65 patients with fistula-associated anal ADC in CD found that 95% of patients had an advanced disease stage at diagnosis (T3–T4), with inguinal lymph node stations involvement in half of the cases and a 10% rate of systemic metastasis [57]. 

These data indicate that APR alone is not curative in a considerable number of patients with ADC in CD: chemotherapy and radiation should be used in addition to surgery in a neoadjuvant or adjuvant setting [55,57]. 

While there are no guidelines for treatment selection in patients with fistula-associated ADC in the setting of CD, general treatment principles for SCC in CD are similar to those in patients without perianal CD. 

Until the mid-1980s, radical surgery with abdominoperineal resection (APR) represented the cornerstone of treatment [180,181] for locally advanced squamous anal cancer, but still being associated with high risk of recurrence and a 40–70% 5-year survival rate [182]. 

In 1974, the Nigro protocol (combination of chemotherapy with 5 fluorouracil and mitomycin and radiation) demonstrated a complete tumor response with the same rate of overall and disease-free survival as surgery, plus sphincter preservation [183,184]. Several subsequent randomized controlled trials [185,186,187,188,189] managed to demonstrate higher disease-free survival and a decreased local failure and recurrence rate for the combined radiation and chemotherapy over radiation alone. 

As a consequence, combined chemoradiation represents nowadays the standard of care for patients with SCC due to a 5-year survival advantage [190], while APR is performed as salvage surgery in case of persistent or recurrent disease (counting for 20–30% of patients) [191].

Special considerations are needed for the IBD population: while chemotherapy does not represent a major issue, radiation therapy may be poorly tolerated in an active proctitis setting and in perianal disease. These led multiple authors to advocate upfront surgery as treatment of choice [60,61]. 

Moreover, patients diverted for CD (with a residual anorectum) must be taken into due consideration, because of the likely higher risk for anal cancer [192,193] as the entity of perianal symptoms is reduced. As a consequence, Ogawa et al. suggested considering prophylactic perineal resection in patients with severe perianal CD, while performing simple diverting procedures for patients who are not fit to tolerate major surgical resections [58].

In conclusion, to date the best approach seems to individualize the treatment selection after a case-by-case analysis in a multidisciplinary setting, also taking into account the ability of the patient to tolerate chemoradiation. Further studies are needed to define the best treatment algorithm, as well as the implementation of surveillance protocols to detect anal cancer in CD patients at an early stage.

## 6. Oncological Treatment in CD Patients with Cancer Diagnosis: Special Considerations 

Little evidence about the interactions between chemotherapeutic agents and therapies for IBD are currently present in the literature [194]. Axelrad et al. evaluated cancer treatment safety and outcomes in colorectal cancer patients, both with and without IBD. Colorectal cancer patients with IBD experienced more treatment delays related to gastrointestinal toxicity than patients without IBD (74% vs. 44%, *p* = 0.03). Among patients with stage IV colorectal cancer, the 5-year overall survival was significantly shorter in patients with IBD in comparison to patients without IBD (19% vs. 50%, *p* = 0.017), and a possible explanation could be the higher rate of treatment delays [195]. For instance, the use of chemotherapeutic agents for gastrointestinal cancers combined with IBD therapeutic schemes may lead to multiple and potentially detrimental effects. Specifically, the oral 5-aminosalicylates (5-ASAs) and sulfasalazine may increase nephrotoxicity in case of platinum-based regimens. On the other hand, methotrexate interacts with 5-fluorouracil (5-FU) by suppressing T-cell function [194]. Biological drugs, such as TNF-alpha antagonists combined with fluoropyrimidines, may cause the development or worsening of heart failure, due to a synergic effect [196]. Furthermore, infliximab is associated to an immune-mediated neuropathy that can worsen the typical neurotoxicity of platinum-based chemotherapy [197]. The administration of anti-VEGF drugs (e.g., bevacizumab) is associated with intestinal perforation [198]; therefore, it should be used with great attention in CD patients. Tiersten et al. evaluated the safety of 5-FU-based chemotherapy in patients with IBD and gastrointestinal cancers, reporting an increased risk of severe diarrhea independently of patient age, 5-FU schedule, concomitant radiation or IBD type [199]. A small retrospective study on eight gastrointestinal cancer patients with IBD confirmed diarrhea as the most common adverse event, with 38% of patients experiencing more than seven stools per day over baseline and/or fecal incontinence [117].

On the basis of such considerations, regimens associated to a high risk of gastrointestinal toxicity, such as combinations of fluoropyrimidines plus irinotecan and/or oxaliplatin, anti-epidermal growth factor receptor (EGFR) monoclonal antibody (cetuximab and panitumumab) plus chemotherapy, should be carefully administered. Thus, recent studies evaluating combinations associated with a high risk of diarrhea, such as FOLFOXIRI plus cetuximab or panitumumab, in metastatic colorectal cancer patients, consider IBD as an exclusion criterion [19,200]. 

Regarding immunotherapy treatment of cancer patients with IBD, data are lacking. Indeed, patients with active autoimmune disease and/or receiving systemic steroid therapy or any other form of immunosuppressive therapy were excluded from immunotherapy studies [201,202]. 

On the contrary, few studies demonstrated a benefit on the IBD course from chemotherapy in cancer patients, in particular for those with no-gastrointestinal cancers [19], as previously reported in Section 4.1. In line with Axelrad’s study, Koc et al. [203] described a milder course and a higher remission rate of active IBD disease in cancer patients (only 44% with gastrointestinal cancer) during chemotherapy, with a reduction in IBD medications comparing a time span of 5 years after and before chemotherapy (mesalazine 47% vs. 71%, *p* < 0.01; corticosteroids 9% vs. 32%, *p* = 0.02; anti TNF-alpha 0% vs. 15%, *p* = 0.25; thiopurines 12% vs. 34%, *p* = 0.13).

On the basis of the above considerations, oncologists should consider the risk/benefit ratio in the treatment choice of patients with IBD and cancer, in particular gastrointestinal cancer. In the decision-making, oncologists must take into account not only the possible interactions between cancer and IBD treatments, but also patients’ clinical conditions, age, comorbidities and expectations. Future prospective studies investigating the effects of chemotherapy in patients with IBD may help to identify the most appropriate regimens in order to maximize cancer treatment benefits and avoid unnecessary toxicity. Thus, a multidisciplinary approach between oncologists, gastroenterologists and surgeons is needed to reach the optimal care of cancer in patients with IBD.

## 7. Future Perspectives on IBD Management 

### 7.1. Artificial Intelligence and Machine Learning in IBD and Gastrointestinal Cancer

Artificial intelligence (AI) is a branch of the interdisciplinary field, particularly focused on the development of machines able to perform tasks that can imitate human intelligence. Machine learning (ML) [204] is a subdomain of AI that deals with the development of algorithms that learns to perform a task through experience (i.e., data) without being explicitly programmed to do so. Recent developments in AI and ML research allowed their applications both in surgery and gastroenterology [205,206,207,208,209]. These innovative approaches could represent a step forward in the IBD diagnosis and management, especially for the capability to analyze and make predictions on wide, unstructured datasets. As of today, the main AI and ML applications in the field of IBD are focused on the prediction of treatment response and the early detection of CRC and dysplasia. Using ML algorithms, different authors were able to predict the efficacy of thiopurines, vedolizumab and infliximab in IBD patients [210,211]. Furthermore, the introduction of integrated AI-assisted detection models could improve conventional colonoscopy [212,213,214]. In particular, as reported by Abadir et al. [215], AI can enhance the detection rate of adenoma in the general population, with a sensitivity and specificity of 98% and 93%, respectively. Additionally, the use of ML could help to distinguish between different types of polyps (benign or malignant) and between inflammatory or neoplastic lesions [216]. 

### 7.2. The Role of Microbiota in IBD and Gastrointestinal Cancer Development 

A well-established relationship between gut microbiota alterations and CD has been documented in the last few years; it has been observed that different microbial species can initiate and promote inflammation, as well as exert anti-inflammatory properties [217]. Interestingly, a link between microbiota alterations and colitis-associated CRC has also been highlighted [218]. In particular, the “common ground hypothesis” has been advanced, according to which barrier disfunction leads to gut dysbiosis, thereby triggering chronic inflammation and, potentially, malignant transformation [219]. Specific microbial species have been associated with CRC: for instance, a significant expansion of *Fusobacterium nucleatum*, *Escherichia coli* and *Bacteroides fragilis* has been reported to be expanded in patients with CRC [218]. Interestingly, it has been speculated that specific CRC-associated microbial signatures might be exploited as a screening tool for intestinal cancer in the future [220]. Human gut microbiota can shape the immune response and, thus, exert pro-carcinogenic effects in two ways: by sustaining chronic inflammation and by reducing the ability of the immune system to counteract malignant transformation. An interesting case could be made for lipocalin-2 and IL-10 knock-out mice, who develop spontaneous colitis and CRC: it was observed that inflammation, and therefore tumorigenesis, could be abolished by antibiotic therapy and, conversely, that the colitis phenotype is transmissible to wild-type mice by crossfostering them with experimental mice [221]. Given the above considerations, it has been speculated that microbiota manipulation could be exploited in cancer treatment to exert direct anti-carcinogenic effects [222] or to enhance the efficacy of cancer therapy [223].

## 8. Conclusions

The diagnosis and treatment of gastrointestinal cancers in CD patients still represent a relevant challenge. An adequate evaluation of the risk factors, ad hoc surveillance and choosing the most appropriate treatment strategy are key factors for the long-term outcomes of these patients. This further underlines the importance of multidisciplinary management in such a challenging clinical condition. Further studies, in a randomized setting and involving a large sample size, are still needed to better define the best diagnostic and treatment pathways.
